# Geometric morphometrics as a tool to understand biogeographical and evolutionary patterns in crane fly genus *Ischnotoma* Skuse (Diptera, Tipulidae)

**DOI:** 10.7717/peerj.13123

**Published:** 2022-03-17

**Authors:** Jéssica Gouvêa, Leonardo H. Gil-Azevedo

**Affiliations:** Departamento de Entomologia, Universidade Federal do Rio de Janeiro, Museu Nacional, Rio de Janeiro, Rio de Janeiro, Brazil

**Keywords:** Canonical Variate Analysis, Principal Components Analysis, Taxonomy, Tipulomorpha, Regression Analysis, Regression Residual Analysis

## Abstract

**Background:**

The geometric morphometric analysis is applied for the first time for the family Tipulidae to distinguish evolutionary and biogeographical patterns on *Ischnotoma* species from Neotropical and Australian regions. We included 45 recognized species of the genus, representing its three subgenera *I*. (*Icriomastax*), *I.* (*Ischnotoma*), and *I*. (*Neotipula*). This paper aims to test if the three subgenera are recoverable using this set of morphometric data.

**Methods:**

Twenty-two landmarks were selected on the wing, mostly located on the radial and medial veins. A Regression Analysis, Principal Components Analysis (PCA), a Shape Coordinates PCA and a Canonical Variate Analysis (CVA) were used to test the variations among individuals, wing shape and groups.

**Results:**

For all analyses, the species of *Ischnotoma* (*Neotipula*) has a strong dissociation from the remaining species and the CVA shows a complete separation of the three subgenera. This study represents the first insight for a new assessment of *Ischnotoma* and the first step to giving a possible new status for *I*. (*Neotipula*).

## Introduction

**Figure 1:**
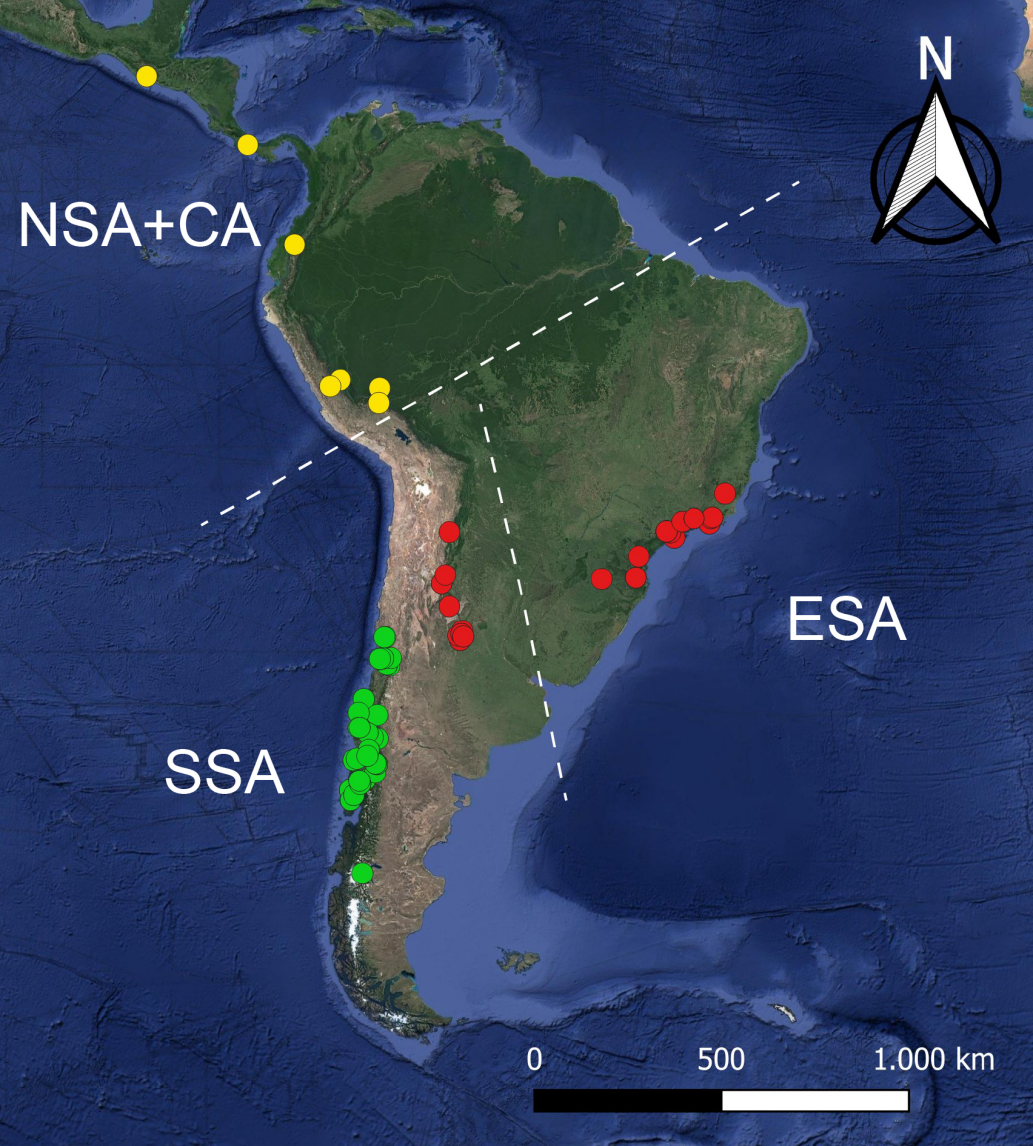
Satellite image of the Neotropical region presenting all localities known for *Ischnotoma*. The dots represent the distribution of *I.* (*Icriomastax*) (red circles), *I.* (Ischnotoma) (green circles) and *I. Neotipula*) (yellow circles) and divided into Eastern South America (ESA), Southern South America (SSA) and Northwestern of South America + Central America (NSA+CA).

**Figure 2:**
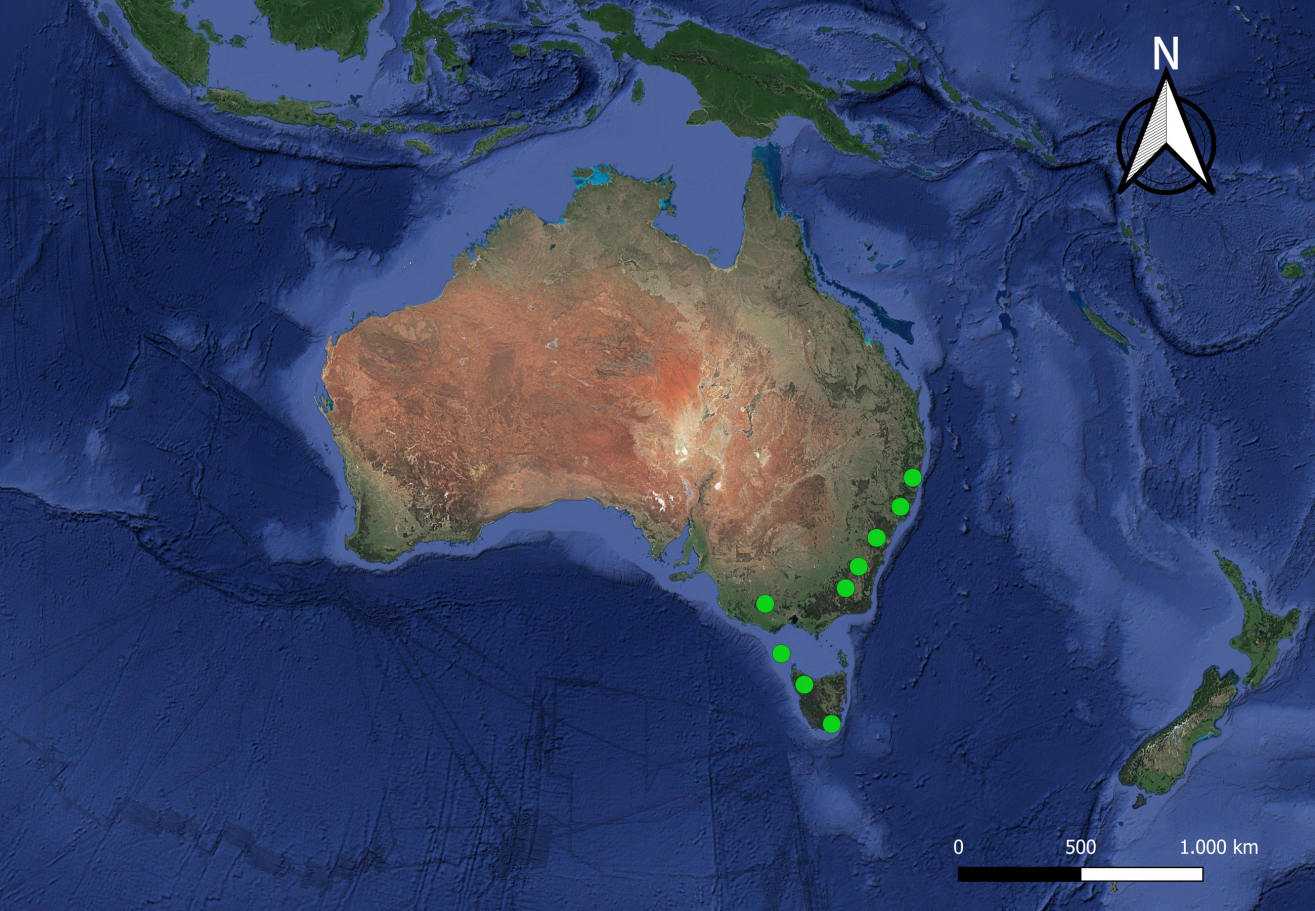
Satellite image of the Australian region. The dots represent the distribution of all localities known for *I.* (*Ischnotoma*) species (green circles).

*Ischnotoma* Skuse, 1890 includes three subgenera, *I*. (*Icriomastax*) Enderlein, 1912, with ten species from Brazil and Argentina; *I.* (*Ischnotoma*) Skuse, 1890, with 35 species distributed on the south of South America and Australia; and *I*. (*Neotipula*) Alexander, 1940, with four species occurring from Peru to Guatemala (Oosterbroek, 2021) and has as type species *I.* (*Ischnotoma*) *eburnea* (Walker, 1848). Figures 1 and 2 shows the current distribution for the genus, where most of the species are only known for their type locality. Some of the diagnostic characters for the genus are the antenna primitively filiform or modified, either produced below or with the last four or five segments sharply reduced in diameter, veins R_4_ and R_5_ in some cases curved towards each other at their midlength, vein Rs longer than m-cu but subequal in *I*. (*Icriomastax*) and squama commonly bare, but when haired not forming a well-defined tuft (Vane-Wright, 1967).

Vane-Wright (1967) reassessed the genus and discussed morphological characters, especially in comparison to the other two close related genera, *Holorusia* Loew, 1863 and *Zelandotipula* Alexander, 1922a. In his conclusion, *Zelandotipula* is not closely related to *Holorusia* and *Ischnotoma* and these two genera may form a sister group. Also, the author considered that *Ischnotoma* possibly reached South America and Australia originally from the North. Vane-Wright (1967) did not consider the *I*. (*Neotipula*) species because, by then, they were placed in the genera *Holorusia* and *Tipula* Linnaeus, 1758. Alexander & Alexander (1970) transferred the four species to *Ischnotoma* and created a new subgenus for them. However, the authors did not explain their choice.

Dobrotworsky (1971) reviewed the 15 species from Australia, provided a key, and separated them into three groups: the *eburnea* group, seven species, the *rufiventris* group, seven species, and the *skuseana* group, with only one species. Therefore, the Neotropical and Australian species of *I.* (*Ischnotoma*) were never studied to investigate the relationship of the species in the subgenus

*Ischnotoma* and other crane fly genus have been included in southern hemisphere biogeographic studies (Ribeiro & Eterovic, 2011; Ribeiro et al., 2014). Those studies showed the great potential of crane flies for biogeography research, considering its worldwide distribution, large number of species and extensive fossil record (Ribeiro & Eterovic, 2011). *Ischnotoma* is not the only crane fly group to share this interesting distribution. Ribeiro & Eterovic (2011) reveal a clear pattern in which crane fly taxa shared between southern South America, New Zealand and Australia and that about 700 species, including *Ischnotoma* species, occur in both South America and Australasia and only in these areas.

Geometric morphometrics is a method where instead of measurements of distances between landmarks, it uses coordinates of landmarks (Villegas, Feliciangeli & Dujardin, 2002) that allows us to visualize differences among complex shapes (Zelditch, Swiderski & Sheets, 2004). Geometric morphometries allow us to recognize features of organisms that we may previously not have noticed by providing analytical and graphical means to decompose variation into components that reflect differences at different spatial scales (Naylor, 1996).

Regarding the body parts studies by morphometric analyses, wings were the major target in Diptera studies (Tatsuta, Takahashi & Sakamaki, 2018). Several families of Diptera have been used as models to geometric morphometrics methods such as Simuliidae (*e.g.*, Pepinelli, Spironello & Currie, 2013; Pepinelli & Currie, 2017), Diopsidae (*e.g.*, Worthington, Berns & Swallow, 2012), Tephritidae (*e.g.*, Dias et al., 2016) and Syrphidae (*e.g.*, Milankov et al., 2013; Nedeljković et al., 2013). However, this method has never been used with any Tipuloidea family before.

In landmark-based studies, variables are not selected *a priori,* they are discovered by the analysis (Zelditch, Swiderski & Sheets, 2004). Its methods use all the information available about the locations of those landmarks while rigorously adhering to the geometric definition of shape (Bookstein, 1996). Examples of very used methods are Principal Components Analysis, Shape Coordinates PCA and Canonical Variates Analysis. The former two are used to explore the shape variations (such as in wing, body or genitalia) among the species. The latter can be used to investigate the variation among groups, in our case the subgenera.

The morphology of *I*. (*Neotipula*) species seems to be the more questionable among the *Ischnotoma* genus. The fact that its species previously belonged to contrasting genera (*Holorusia* and *Tipula*), suggests the hypothesis that this subgenus may not be a true *Ischnotoma.* Therefore, this work aims to test the use of geometric morphometrics on Tipulidae species, investigating the impacts of different wing shapes to provide insights into whether *Ischnotoma* species belong to three separate groups, how closely related they might be, and if Neotropical and Australian species can be discriminated into two separate groups based on their distribution.

## Materials & Methods

All species of *I*. (*Icriomastax*) and *I*. (*Neotipula*), and 31 species of *I*. (*Ischnotoma*) (of the 35), were included in this analysis. Most of the material used here represent holotypes, allotypes and paratypes (Supplemental Information 2).

**Figure 3:**
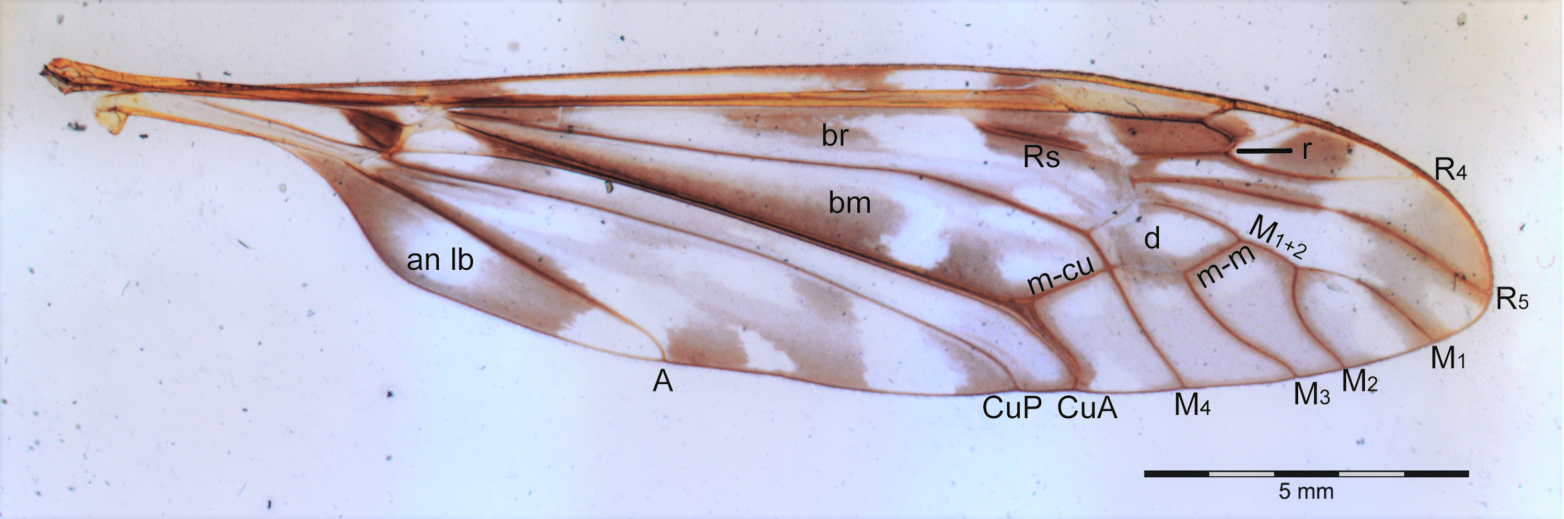
Wing of *I.* (*Ischnotoma*) *eburnea* (Walker, 1848).

All analyzed specimens are deposited at the Entomology Collection of the National Museum of Natural History (Smithsonian Institute) in Washington, DC, USA, except the holotype of *I*. (*Ic.*) *nudicornis* (Macquart, 1838), which is housed at the Natural History Museum in London, UK. The slide images were taken with an Olympus DP 80 camera. For the six species with no preserved wings on slides, *I*. (*Ic.*) *nudicornis*; *I*. (*Is.*) *araucana* (Alexander, 1929); *I*. (*Is.*) *concinna* (Philippi, 1866); *I*. (*Is.*) *decorata* (Philippi, 1866); *I*. (*Is.*) *rufistigmosa* (Macquart, 1838), and *I*. (*Is.*) *silvai* (Alexander, 1929), an Olympus DSX 100 camera were used to photograph the adult specimens. Even though there is a compression to flatten the wings on slides, no distortion of measurement compared to unmounted wings were observed. The venation terminology follows De Jong (2017) (Fig. 3).The maps were made using the software QGIS 3.18 (QGIS Development Team, 2021) and satellite images from Google Earth Engine^®^ (Gorelick et al., 2017).

**Figure 4:**
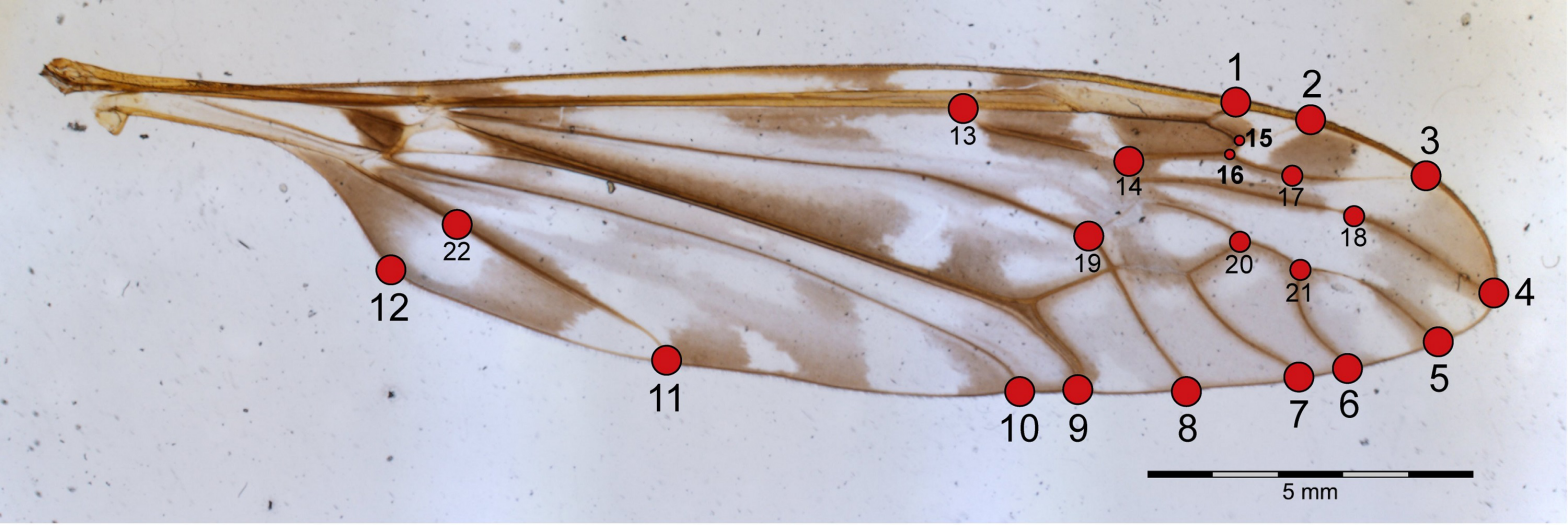
Wing of *I.* (*Ischnotoma*) *eburnea*. The dots represent the landmarks used on the geometric morphometrics analysis.

There is no previous literature suggesting the position of landmarks on Tipulidae wings for morphometric studies. Therefore, the choosing of the landmarks followed our own observations of the genus, considering diagnostic venation characters, but with two basic assumptions: all landmarks had to be homologous for all species of the genus and they must represent the points with high variation between the species. Twenty-two landmarks were selected on the wing, comprising most of the radial and medial veins (Fig. 4). The landmarks 1 to 11 represent the point where the veins reach the margin of the wing. Landmark 12 represents the apex of the anal lobe, which could be very developed, as in *I*. (*Neotipula*), or less curved, as in *I.* (*Is.*) *tarwinensis* Alexander, 1928. Landmarks 13 and 14 indicate the length of vein Rs, which could be long, exceeding twice the length of m-cu, as in *I*. (*Is.*) *larotypa* (Alexander, 1929), or shorter, not exceeding twice the length of m-cu, as in *I*. (*Is.*) *skuseana* Alexander, 1928a; Alexander, 1928b. Landmarks 15 and 16 represent the length and position of crossvein r, since it joins vein R_3+4_ in *I*. (*Neotipula*), or vein R_3_, as in *I*. (*Icriomastax*) and *I*. (*Ischnotoma*). Landmark 17 was placed at the lowest point of vein R_4_ and landmark 18 at the highest point of vein R_5_, both to represent the curve of the veins. However, *I*. (*Is.*) *episemafrom* Alexander, 1924a, *I*. (*Is.*) *immaculipennis* Alexander, 1924a, *I*. (*Is.*) *prionoceroides* Alexander, 1922b, *I*. (*Is.*) *scutellumnigrum* Alexander, 1924a, and all *I*. (*Neotipula*) species shows a nearly straight vein R_4_. For those, landmark 17 was placed at mid-length of the vein. The landmarks 19 and 20 show the length variation of the discal cell, elongated, as in *I*. (*Is.*) *tarwinensis*, or reduced, as in *I*. (*Is.*) *penai* (Alexander, 1952). Landmarks 20 and 21 represent the length of vein M _1+2_, that could be shorter than m-m as in *I*. (*Is.*) *rubriventris* (Macquart, 1846) or longer than m-m as in *I*. (*Is.*) *rufiventris* (Macquart, 1846). Landmark 22 was placed at the closest distance between landmark 12 and vein A, to indicate the width of the anal lobe.

The landmarks were scaled using the tps-Dig software (Rohlf, 2010). We performed the Generalized Procrustes Analysis (GPA) where every landmark is translated, scaled, and rotated iteratively until the least fit of all landmarks to a consensus is no longer improved (Rohlf & Slice, 1990). The centroid size, a measure independent of shape, for each species was obtained using the PAST software (Hammer, Harper & Ryan, 2001). The Principal Component Analysis (PCA) was executed to quantify the shape variations between species. A Shape Coordinates PCA, equivalent to PCA, was made on tps Relw software to study the variation in shape of the specimen’s wing and for better visualization of the variation. A Regression analysis was made to investigate shape and size separately with a permutation test with 10.000 rounds and *P*-value of 0.0007. We also performed a Regression Residual Analysis which exclude the information of size over shape.

A Canonical Variate Analysis (CVA) was performed to associate the variations in interspecific shapes (Manly & Alberto, 2016) with permutation test for pairwise Mahalanobis distances set for 10,000 rounds. For the CVA two runs were made, the first one considering only the main three groups, *I*. (*Icriomastax*), *I*. (*Ischnotoma*), and *I*. (*Neotipula*), and the second separating the specimens according to biogeographical distribution, Australian or Neotropical. For better understanding, the Neotropical distribution was divided into three subgroups: Eastern South America (ESA), including most of *I*. (*Icriomastax*) species; Southern South America (SSA), with *I*. (*Ischnotoma*) species and two *I*. (*Icriomastax*) species from Argentina; and Northwestern of South America + Central America (NSA+CA), representing *I*. (*Neotipula*). The Regression, PC and CV analyses were carried out on MorphoJ (Klingenberg, 2011).

## Results

**Figure 5:**
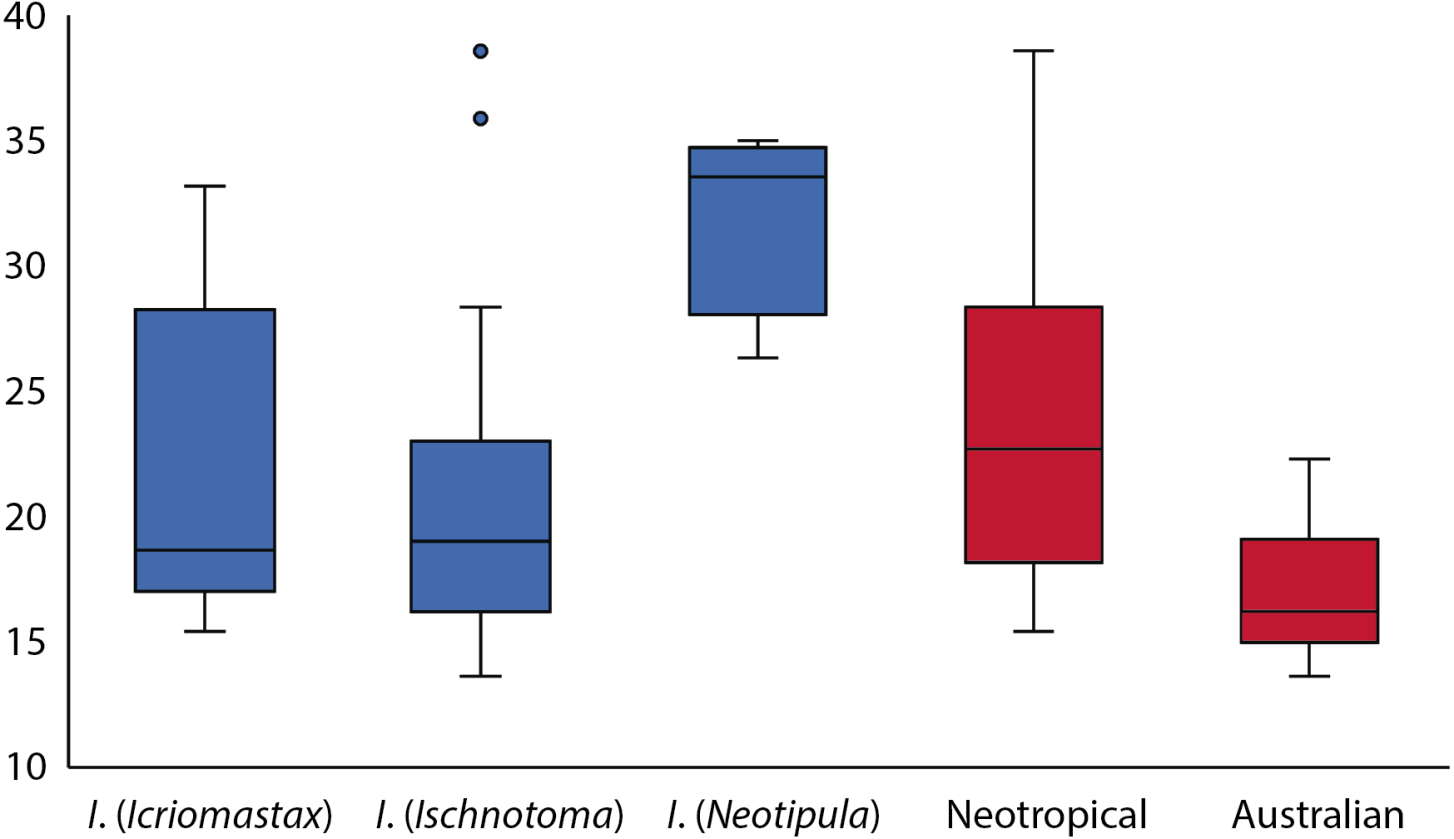
Box plot with centroid size variation. In blue are the variation of the three subgenera and in red the variation according to their distribution.

Table 1 shows the centroid size of all species analyzed and Fig. 5 shows the variation of centroid size between subgenus and distribution areas. The only two argentinians species, *I*. (*Ic.*) *nudicornis* and *I*. (*Ic.*) *ocellata* (Enderlein, 1912), stood out from the subgenus, having the only values over 30 mm. The chileans species *I*. (*Is.*) *araucana* and *I*. (*Is.*) *rufistigmosa* had the highest centroid size for *I*. (*Ischnotoma*), being the outliers on Fig. 5, and finally, the subgenus with highest values is *I*. (*Neotipula*) having three of the four species with centroid sizes over 30 mm. The average centroid size for each subgenera was 21.71 mm for *I*. (*Icriomastax*), 20.25 mm for *I*. (*Ischnotoma*) and 32.10 for *I*. (*Neotipula*). Considering only the distribution, the Neotropical species presented 23.08 mm and the Australian species 16.82 mm average centroid size.

**Table 1:** Centroid sizes for each studied species. The * symbol represents the *I*. (*Ischnotoma*) species within Australia.

**Species**	**Centroid size (mm)**
*I*. (*Icriomastax*) *antinympha* (Alexander, 1942)	26.979
*I*. (*Icriomastax*) *calliope* (Alexander, 1945a)	15.4088
*I*. (*Icriomastax*) *euterpe* (Alexander, 1945b)	17.7015
*I*. (*Icriomastax*) *helios* (Alexander, 1949)	19.1673
*I*. (*Icriomastax*) *jujuyensis* (Alexander, 1920)	17.4284
*I*. (*Icriomastax*) *nitra* (Alexander, 1945b)	18.1734
*I*. (*Icriomastax*) *nudicornis* (Macquart, 1838)	32.032
*I*. (*Icriomastax*) *ocellata* (Enderlein, 1912)	33.1669
*I*. (*Icriomastax*) *phaeton* (Alexander, 1945a)	15.8191
*I*. (*Icriomastax*) *zikani* (Alexander, 1936)	21.2771
*I*. (*Ischnotoma*) a*raucana* (Alexander, 1929)	38.5689
*I*. (*Ischnotoma*) *concinna* (Philippi, 1866)	19.0121
*I*. (*Ischnotoma*) *decorata* (Philippi, 1866)	23.9792
*I*. (*Ischnotoma*) *delpontei* (Alexander, 1929)	18.9764
*I*. (*Ischnotoma*) *eburnea* * (Walker, 1848)	22.3081
*I*. (*Ischnotoma*) *episema* * Alexander, 1924a	19.1514
*I*. (*Ischnotoma*) *fagetorum* (Alexander, 1929)	19.7284
*I*. (*Ischnotoma*) *fastidiosa* * (Skuse, 1890)	16.2067
*I*. (*Ischnotoma*) *fuscostigmosa* (Alexander, 1929)	23.3672
*I*. (*Ischnotoma*) *goldfinchi* * Alexander, 1924b	19.0851
*I*. (*Ischnotoma*) *immaculipennis* * Alexander, 1924a	16.2161
*I*. (*Ischnotoma*) *larotypa* (Alexander, 1929)	26.3869
*I*. (*Ischnotoma*) *par* * (Walker, 1856)	19.8489
*I*. (*Ischnotoma*) *penai* (Alexander, 1952)	18.8052
*I*. (*Ischnotoma*) *peracuta*Alexander, 1971	23.0173
*I*. (*Ischnotoma*) *porteri* (Alexander, 1929)	24.7193
*I*. (*Ischnotoma*) *postnotalis* (Alexander, 1929)	15.4674
*I*. (*Ischnotoma*) *prionoceroides* * Alexander, 1922b	16.9354
*I*. (*Ischnotoma*) *problematica* (Alexander, 1945c)	16.956
*I*. (*Ischnotoma*) *rubriventris* * (Macquart, 1846)	18.2402
*I*. (*Ischnotoma*) *rubroabdominalis* * Alexander, 1922b	15.4528
*I*. (*Ischnotoma*) *rufistigmosa* (Macquart, 1846)	35.875
*I*. (*Ischnotoma*) *rufiventris* * (Macquart, 1846)	15.2947
*I*. (*Ischnotoma*) *schineriana* (Alexander, 1928a)	17.0086
*I*. (*Ischnotoma*) *scutellumnigrum* * Alexander, 1924a	15.5096
*I*. (*Ischnotoma*) *shannoniana* (Alexander, 1929)	19.5111
*I*. (*Ischnotoma*) *silvai* (Alexander, 1929)	28.3539
*I*. (*Ischnotoma*) *skuseana* * Alexander, 1928b	14.0596
*I*. (*Ischnotoma*) *tarwinensis* * Alexander, 1928b	13.642
*I*. (*Ischnotoma*) *terminata* * Alexander, 1928b	13.668
*I*. (*Ischnotoma*) *trunculata* (Alexander, 1962)	22.6866
*I*. (*Neotipula*) *maya* (Alexander, 1912)	26.3238
*I*. (*Neotipula*) *paprzyckii* (Alexander, 1941)	33.2294
*I*. (*Neotipula*) *pectinella* (Alexander, 1940)	33.8661
*I*. (*Neotipula*) *penata* (Alexander, 1966)	34.9991

**Figure 6:**
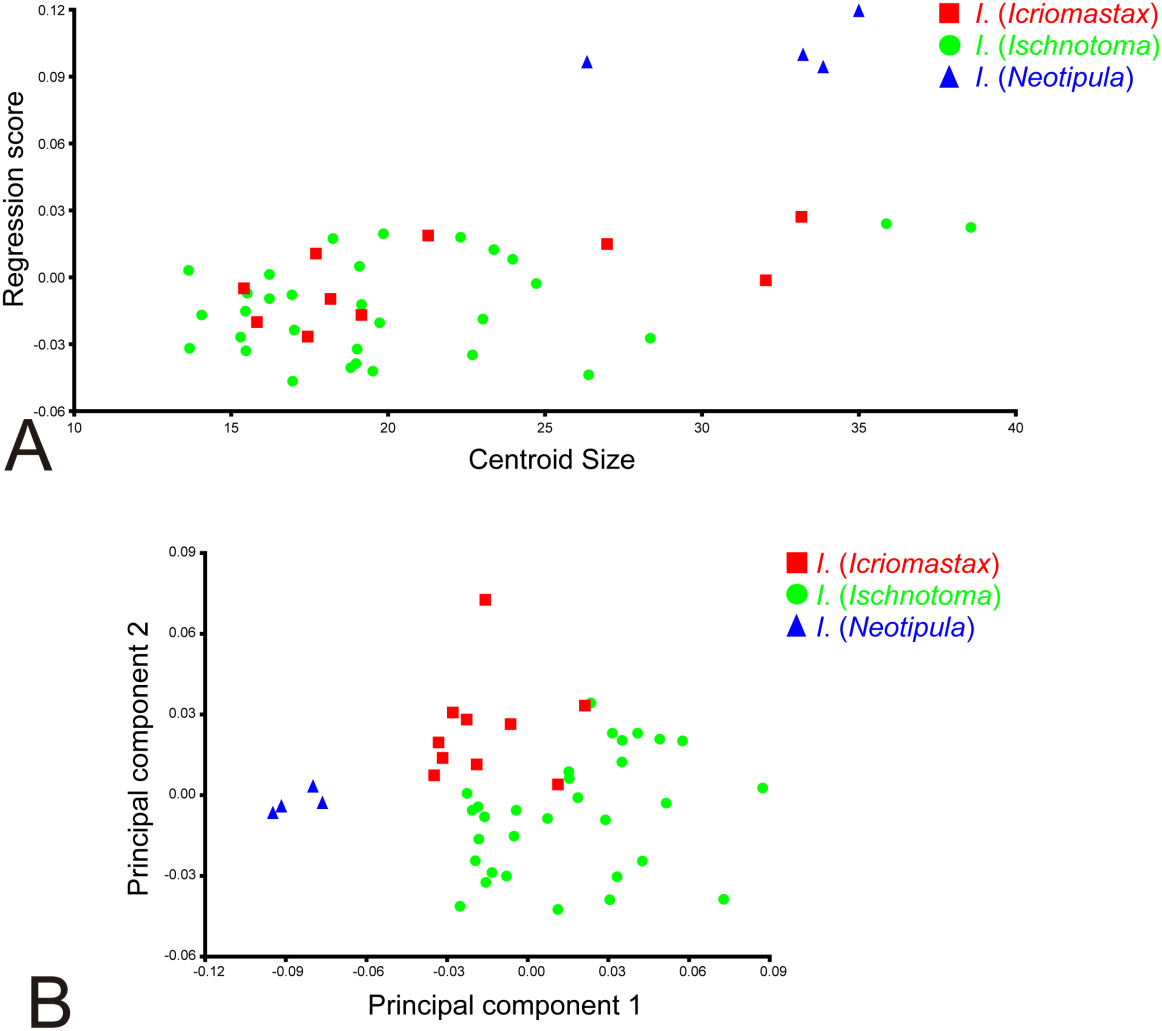
Allometric regression analysis. (A) Regression analysis with two variables, the independent centroid size and the dependent shape, represented by the regression scores; (B) Regression Residual Analysis, excluding all allometric information.

The Regression Analysis showed a correlation between smaller size (the independent variable) and low regression score (*i.e.,* shape, the dependent variable) but not between larger size and high regression score (Fig. 6A). For this data set we found 12.75% of allometry. The Regression Residual Analysis found 67.96% for the first three PCs (PC1 42.72%, PC2 15.37%, PC3 9.87%) with similar dispersion of *I*. (*Icriomastax*) and *I*. (*Ischnotoma*) and *I*. (*Neotipula*) with very distinct scores (Fig. 6B).

**Figure 7:**
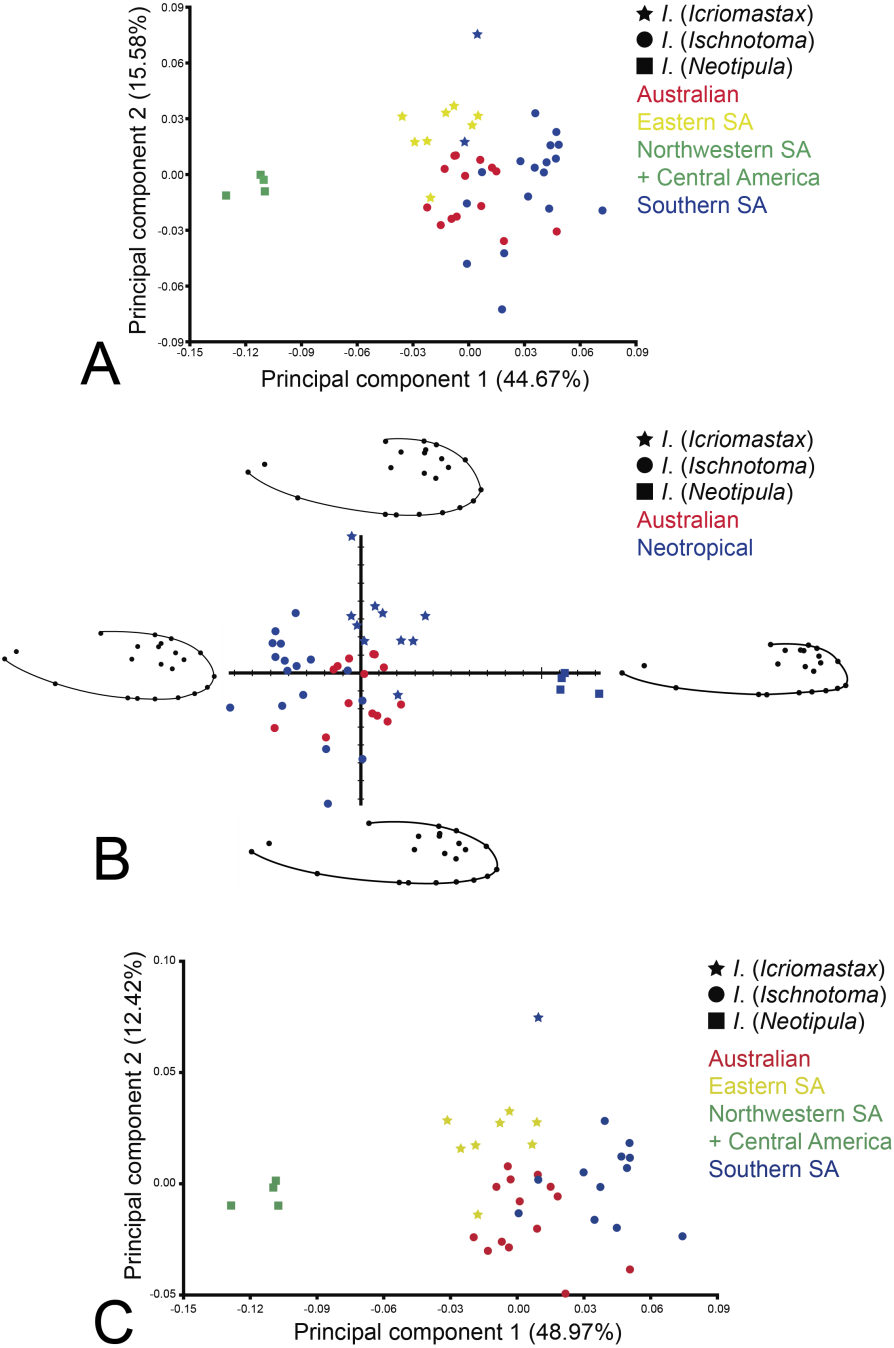
PCA of the two first factors based on scores of 22 landmarks of 45 species of *Ischnotoma*. (A) Dispersion of the species based on subgenera: *I*. (*Icriomastax*) (stars); *I*. (*Ischnotoma*) (circles) and *I*. (*Neotipula*) (squares), and region of occurrence, Australia (red), Eastern SA (yellow), Northwestern SA + Central America (green) and Southern SA (blue), *X*-axis: first principal component (44.67%). *Y*-axis: second principal component (15.58%); (B) Shape Coordinates PCA of *Ischnotoma* species representing the wing shape variation. *I.* (*Icriomastax*) (stars); *I*. (*Ischnotoma*) (circles) and *I*. (*Neotipula*) (squares), and region of occurrence, Australia (red) and Neotropical (blue); (C) dispersion of only slide-mounted specimens based on subgenera: *I*. (*Icriomastax*) (stars); *I*. (*Ischnotoma*) (circles) and *I*. (*Neotipula*) (squares), and region of occurrence, Australia (red), Eastern SA (yellow), Northwestern SA + Central America (green) and Southern SA (blue), *X*-axis: first principal component (48.97%). *Y*-axis: second principal component (12.42%).

The first three components of the PCA accounted very similar scores to the found with the Regression Residual Analysis, with total 68.89% for the first three PCs among the analyzed species (PC1 44.67%, PC2 15.58%, PC3 8.64%) (Fig. 7A). It is expected large difference between the variances of the first two PCs, and much smaller differences between successive pairs of components (Zelditch, Swiderski & Sheets, 2004). Regarding PC1, *I*. (*Icriomastax*) and *I*. (*Ischnotoma*) had similar dispersion along the axis, with scores between −0.03 and 0.08, in contrast to all species of *I*. (*Neotipula*) which formed a completely distinct group placed around −0.10 score. Most species from Neotropical and Australian regions were located around the 0.00 score on the axis and it is possible to indicate *I*. (*Ischnotoma*) as having a higher range within the PC1 axis. Also, most *I*. (*Icriomastax*) species share similar scores with *I*. (*Ischnotoma*) while the latter has most of their species isolated with scores higher than 0.02. The PC2 indicates a higher concentration of species near the 0.00 value at the axis, however, it is not a good variable to separate the subgenera. *Ischnotoma* (*Icriomastax*) and *I*. (*Ischnotoma*) continue to overlap each other but *I*. (*Neotipula*) also has similar PC2 scores. As observed on PC1, some *I*. (*Ischnotoma*) species appear set apart from the main group, but this time with lower scores. However, when separating the species according to distributional records neither PC1 nor PC2 separates the Australian from the Neotropical species (Fig. 7A). The PCA showed a higher dispersion on the PC1 axis, especially due to *I*. (*Neotipula*) species.

Since the equivalence to PCA, the Shape Coordinates PCA plot showed the same results (Fig. 7B). However, with this graph is clearer to visualize the possible wing’s variation according to their shape. The left side of the graph represent the species with an elongate and narrower apex wing. On the right side, we see the *Ischnotoma* species with shorter wing length and round apex. The vertical axis shows differences on width, the upper part the width is wider than the bottom part of the graph. The general shape of the wing also puts *I*. (*Neotipula*) separated from the other species. On the other hand, *I*. (*Icriomastax*) and *I*. (*Ischnotoma*) form a more homogeneous group. Most *I*. (*Icriomastax*) are on the upper part of the graph, representing wider width in comparison to some species of *I*. (*Ischnotoma*). Most species of *I*. (*Ischnotoma*) are on the right side of graph, showing a similar wing length.

Since we included slide-mounted and non slide-mounted specimens, we also did a PCA without the six non slide-mounted specimens to evaluate if this part of the sample could trigger potential differences. This second PCA (Fig. 7C) showed that the absence of the non slide-mounted specimens did not altered the final result. The first three components of the PCA accounted for 70.15% of the variations among the analyzed species (PC1 48.97%, PC2 12.42%, PC3 8.76%).

**Figure 8:**
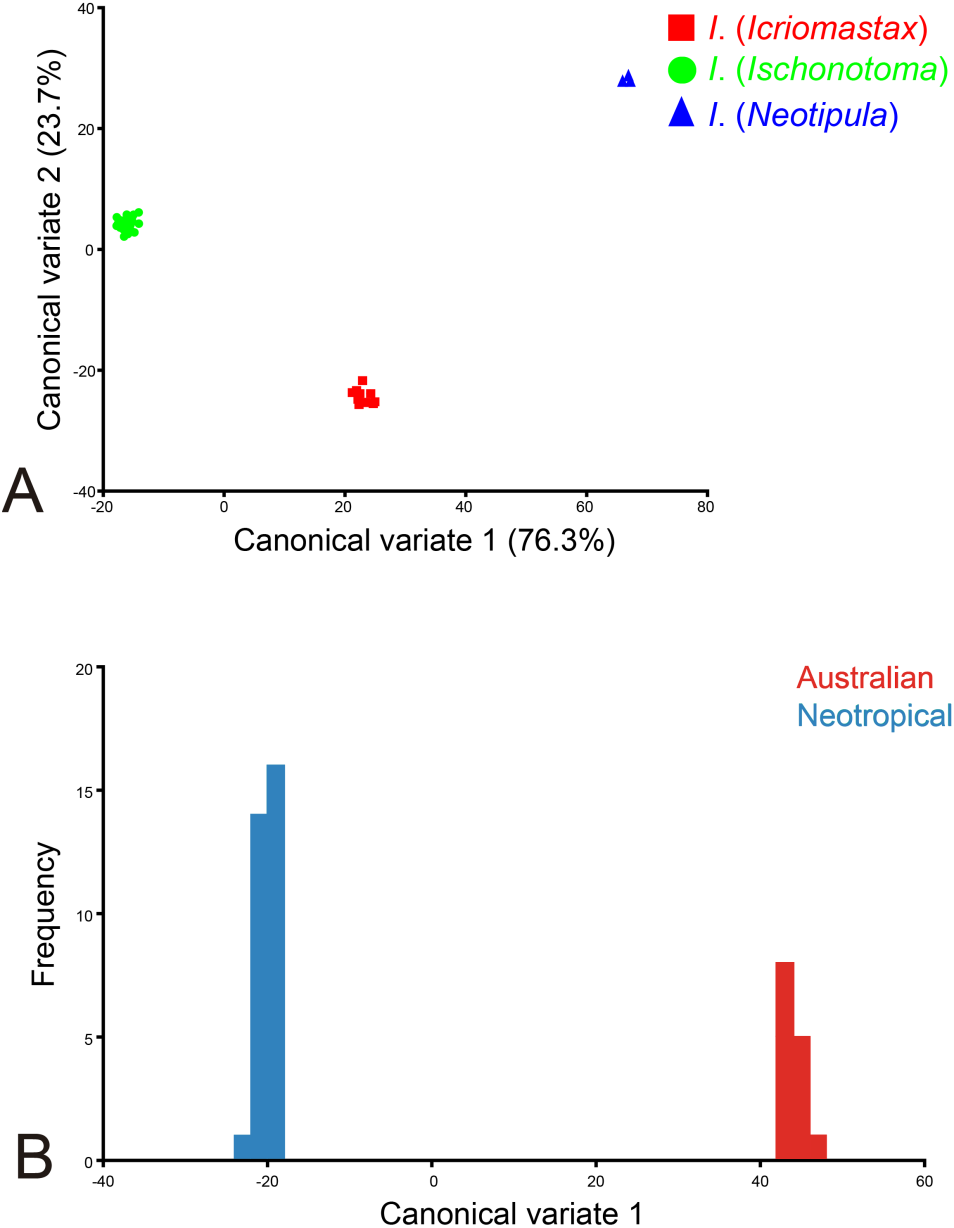
Canonical Variate Analysis. (A) Graphic of the first two canonical variates for *Ischnotoma* species: *I*. (*Icriomastax*) (red), *I*. (*Ischnotoma*) (green) and *I*. (*Neotipula*) (blue) based on 22 landmarks. *X*-axis: first canonical variable (76.3%). *Y*-axis: second canonical variable (23.7%); (B) graphic of the first canonical variable from the pairwise comparison for *Ischnotoma sensu stricto* species from Australian (red) and Neotropical (blue) regions based on 22 landmarks. *X*-axis: first canonical variable. *Y*-axis: frequency.

The CVA showed the three subgenera completely separated. For both CV1 and CV2 axis, *I*. (*Neotipula*) presented the highest scores of the landmarks. *Ischnotoma* (*Icriomastax*) showed higher CV1 and lower CV2 scores in comparison to *I*. (*Ischnotoma*) (Fig. 8A). When considering the distribution with 14 Australian and 31 Neotropical species, the Eigenvalues is 924.18, the Mahalanobis distances among the groups is 64.19 and the *P*-value from permutation tests for Mahalanobis distances among the groups is 0.0007. The Neotropical species showed a higher frequency than the Australian species with CV1 (Fig. 8B).

## Discussion

Centroid size is the one measure of size that is mathematically independent of shape. Empirically, centroid size may often be correlated with shape because larger organisms are usually shaped differently than smaller ones (Zelditch, Swiderski & Sheets, 2004). Large species, as all *I*. (*Neotipula*) and a few *I*. (*Icriomastax*) and *I*. (*Ischnotoma*), naturally have large centroid size. Knowing that centroid size considers only the size, if we correlate with shape we have the basic concept of allometry. According to the Gould–Mosimann school, allometry is characterized as the association between a standard size variable and the corresponding vectors of shape ratios or between a log-size variable and log-shape variables (Klingenberg, 2016).

With the Regression Analysis (Fig. 6A) it is possible to determine if there is any inherent asymmetry between shape (dependent variable) and size (independent variable). As expected, smaller species, with centroid size under 23 mm, has similar shapes, not exceeding 0.03 regression score. However, the same is not applied to the larger species. The larger *I*. (*Icriomastax*) and *I*. (*Ischnotoma*) has similar or higher centroid size of most *I*. (*Neotipula*) but does not have similar regression scores. This means that for *I*. (*Icriomastax*) and *I*. (*Ischnotoma*) the shape variation is not as high as we see for *I*. (*Neotipula*). Therefore, for *I*. (*Neotipula*) the shape effects has more potential to interfere and separate its species from the other subgenera. On the other hand, since the shape is more similar, the size has more influence to separete *I*. (*Icriomastax*) and *I*. (*Ischnotoma*) from *I*. (*Neotipula*), even though most species have centroid size under 25 mm.

When the 12.75% of allometry found with the Regression Analysis is excluded, *i.e.,* when the information of size over shape is no longer considered (Fig. 6B), we have a very similar graph in comparison to the PCA (Fig. 7A). The low percentage of allometry is because most species in the genus, that is the *I*. (*Icriomastax*) and *I*. (*Ischnotoma*) with centroid size under 25 mm, does not have high variation in form and only the four *I*. (*Neotipula*) have a correlation between high centroid size and high regression scores. Thus, when the allometry is excluded we do not find strong differences between this result and the PCA.

The PC analysis is a tool that constructs variables that can be used to examine variation among individuals within a sample (Zelditch, Swiderski & Sheets, 2004). The utility of PCA lies in the fact that many (if not all) of the features measured in a study will exhibit covariances because they interact during, and are influenced by, common processes. (Zelditch, Swiderski & Sheets, 2004). With the PCA our aim in this study was to see the range of variation among the individuals, without specifying different groups, in this case, subgenera. Since *I*. (*Icriomastax*) and *I*. (*Ischnotoma*) species are closer to each other and all *I*. (*Neotipula*) species are together and distant from the remaining species, we can assume that *I*. (*Icriomastax*) and *I*. (*Ischnotoma*) species have less variation among each other than in comparison with *I*. (*Neotipula*), specially according to PC1. In other words, within *Ischnotoma*, we found two ranges of variation among the species: the first one isolates *I*. (*Neotipula*) and the second includes both *I*. (*Icriomastax*) and *I*. (*Ischnotoma*).

The Shape Coordinates PCA shows the distribution of shapes, in this case of the first two principal components, as a direction of shape change about the mean form. Regarding shape variation between the species, the Shape Coordinates PCA demonstrate, as expected, the same strong separation of *I*. (*Neotipula*) and detected an association between *I*. (*Icriomastax*) and *I*. (*Ischnotoma*) as seen in PCA. The first PC (44.67%), represented by the horizontal axis, show the possible variations of wing length, elongate to short, and apex shape, narrow to round. The second PC (15.58%), represented by the vertical axis, show the width variantion, that could be wider or narrow (Fig. 7B).

For *I*. (*Icriomastax*) and *I*. (*Ischnotoma*), the length of the wing showed more influence to put them closer. On the other hand, the same variant is responsible to set *I*. (*Neotipula*) apart. These species have much larger body size than the other subgenera and consequently larger wing size, very similar to many *Holorusia* species, as *H. hespera* Arnaud & Byers, 1990 and *H. clavipes* (Edwards, 1921). However, on the PC2 axis, we do not see a strong separation of *I*. (*Ischnotoma*), since the species present a large range of wing width.

The second PCA (Fig. 7C), with only slide-mounted specimens, was made to make sure there was no bias affecting the groups. The two graphs (Figs. 7A, 7C) shows very similar distribution and PC values, confirming that the mix of slide-mounted and non slide-mounted specimens did not compromise the results.

The CVA is used for simplifying descriptions of differences between groups (Zelditch, Swiderski & Sheets, 2004) so here, we were searching for evidence that proves if *Ischnotoma* really have three subgenera. This analysis shows a strong separation of the three subgenera (Fig. 8A). Since both *I*. (*Icriomastax*) and *I*. (*Ischnotoma*) have Neotropical species, this strong discrimination between them is an important result because even with their close distribution, they are two different subgenera. Also, *I*. (*Neotipula*) is once again distant from the other subgenera. Considering the distributional records and placing all Neotropical species together, the CVA also separates the Neotropical from Australian species (Fig. 8A). This shows that even with a subgenus on both regions, was not enough to mix the species and both groups, Neotropical and Australian, have enough differences to set them apart.

*Ischnotoma* (*Neotipula*) appears completely set apart on all analyses (Figs. 6, 7, 8) which demonstrates that those species probably have more differences than similarities with the other *Ischnotoma* species. Features showed by the PCA with importance for discriminating this group are the large wing size, a very developed anal lobe and the crossvein r joining vein R_3+4_. These characters are shared with most *Holorusia* species and not with any other species of *Ischnotoma*. These and other similarities are already pointed out by Vane-Wright (1967). Even though the body size was not included in the analysis, it is important to emphasize the allometric characteristics, relevant when it comes to *I*. (*Neotipula*). The species of this subgenus can reach 40 mm of body length, contrasting with the much smaller *I*. (*Icriomastax*) and *I*. (*Ischnotoma*) species. Allometry is a common phenomenon, so we might expect that size and shape would usually be statistically correlated (Zelditch, Swiderski & Sheets, 2004). Based on the characters presented here, it is possible that the clear dissociation from the *Ischnotoma* species could represent strong evidence that *I.* (*Neotipula*) needs a review and perhaps receive a new status.

*Ischnotoma* (*Icriomastax*) shows close relation to *I*. (*Ischnotoma*) in PC analysis (Fig. 7A). However, two species appear further away from the remaining species. *Ischnotoma* (*Icriomastax*) *jujuyensis* (Alexander, 1920) from Argentina has the higher PC2 scores (0.09) and it is distant from all other Eastern SA species, including *I*. (*Icriomastax*) *nudicornis* also from Argentina. *Ischnotoma* (*Icriomastax*) *ocellata* groups closer to *I*. (*Ischnotoma*) than to the rest *I.* (*Icriomastax*) (PC1 −0.02; PC2 −0.02). Off all *I.* (*Icriomastax*) species, *I.* (*Icriomastax*) *ocellata* is the more distinct with a larger wing size and this feature may contribute to its distance from the remaining *I.* (*Icriomastax*).

Regarding *I*. (*Ischnotoma*), neither PCA nor CVA separate the Australian species from the South American species (Southern + Eastern + [Northwestern +Central America]). The PC analysis shows most of them overlapping both on PC1 and PC2, respectively around −0.02 and 0.05 and between −0.04 and 0.04 (Fig. 7A) and the CVA clustered all South American and Australian species close together in one group (Fig. 8A).

Hennig (1966) discussed biogeographical hypotheses for *Ischnotoma* and believed the whole group has originated in the Northern Hemisphere and then migrated separately to South America and Australia. Vane-Wright (1967) agrees with Hennig (1966) and states that they may be vital intermediates between Australian and South American species. However, it is important to consider that the authors were dispersalist, thus they did not use other events to help interpret the distribution patterns.

The study of south hemisphere biogeography refers to the feasibility of alternative explanations for the biotic links between southern South America, New Zealand, Australia and the biogeographically related areas of New Guinea and New Caledonia (Ribeiro & Eterovic, 2011). Regarding South American Tipulidae, Alexander (1929) already stated the resemblance of the fauna from Chile and Patagonia to the Australasian fauna. More recently, Ribeiro et al. (2014) included all Tipulidae described species to search for endemism patterns in a worldwide scale. Two of the nine areas of endemism found by them are in the Neotropical and Australian regions, same areas of *Ischnotoma* distribution. Therefore, the interesting current *Ischnotoma* distribution together with the many biogeographical methods available, ones that includes other concepts as vicariance and continental drift, are very important to help understanding distributional patterns of crane flies in the southern hemisphere as evidence of direct evolutionary links between the southern continents (Ribeiro et al., 2014).

## Conclusions

Geometric morphometrics have never been applied to Tipulidae species or any related Tipuloidea family before and here has proved to be a powerful tool to demonstrate the separation of *Ischnotoma* species groups. Even including one specimen per species, our analyses showed important results since our focus was on investigating the possible differences among the whole genus and not between species or populations. Considering all analyses, *I*. (*Ischnotoma*) and *I*. (*Icriomastax*) are closely related but can be separated into groups, forming two distinct subgenera. *I.* (*Neotipula*) on the other hand, needs a deep review to assess the placement of its species and possibly give them a new status.

## Supplemental Information

10.7717/peerj.13123/supp-1 Supplemental Information 1 MorphoJ project

10.7717/peerj.13123/supp-2 Supplemental Information 2 Examined material

## Abbreviations of morphological terms used in text and/or figures

A: anal vein
an lb: anal lobe
bm: basal medial cell
br: basal radial cell
CuA: anterior branch of cubital vein
CuP: posterior branch of cubital vein
d: discal cell
M_1_: first branch of media
M: branch of first and second media
M_2_: second branch of media
M_3_: third branch of media
M_4_: fourth branch of media
m-cu: medial-cubital crossvein
m-m: medial crossvein
r: radial crossvein
Rs: radial sector
R_4_: upper branch of third branch of radius
R_5_: lower branch of third branch of radius

## Additional Information and Declarations

### Data Availability

The following information was supplied regarding data availability: The examined material list is available in a Supplemental File.
