# Supplementary material for: Geometric morphometrics as a tool to understand biogeographical and evolutionary patterns in crane fly genus Ischnotoma Skuse (Diptera, Tipulidae)
Source: PeerJ. 2022 Mar 17;10:e13123. doi: 10.7717/peerj.13123 (PMC8934531; doi:10.7717/peerj.13123)
Supplement: Supplemental Information 2 [file peerj-10-13123-s002.pdf]

Data S1: Examined material.

***Ischnotoma* (*Icriomastax*) *antinympha* (Alexander, 1942).** BRAZIL: 1♀, São Paulo, Juquiá, 400m, xi/1940. L. Travassos leg. (USNMENT 01222590, holotype #7,198).

***Ischnotoma* (*Icriomastax*) *calliope* (Alexander, 1945).** BRAZIL: 1♂, São Paulo, Guarujá, 50m. 21.i.1942. M. Carrera leg. (USNMENT 01222593, holotype #13,091).

***Ischnotoma* (*Icriomastax*) *euterpe* (Alexander, 1945).** BRAZIL: 1♂, Rio de Janeiro, i-iii.1941. Tulloch leg. (USNMENT 01222476, holotype#7,875).

***Ischnotoma* (*Icriomastax*) *helios* (Alexander, 1949).** BRAZIL: 1♂, Paraná, Curitiba, vi.1943. Hatschbach leg. (USNMENT 01222591, holotype#13,092).

***Ischnotoma* (*Icriomastax*) *jujuyensis* (Alexander, 1920).** ARGENTINA: 1♀, Jujuy, 12.i.1920. Weiser leg. (USNMENT 01222587, holotype#1,462).

***Ischnotoma* (*Icriomastax*) *nitra* (Alexander, 1945).** BRAZIL. 1♂, Rio de Janeiro, Teresópolis, 920m, viii.1942, Lério Gomes leg. (USNMENT 01222585, paratype#7,876).

***Ischnotoma* (*Icriomastax*) *nudicornis* (Macquart, 1838).** ARGENTINA: 1♀, Buenos Aires, M. Serville Bigot leg. (BMNH(E)#245194, NHMUK010210571, holotype).

***Ischnotoma* (*Icriomastax*) *ocellata* (Enderlein, 1912).** BRAZIL. Santa Catarina, Nova Teutonia, 29.xi.1938. Plaumann leg. (USNMENT 01222718, #3380).

***Ischnotoma* (*Icriomastax*) *phaeton* (Alexander, 1945).** BRAZIL: 1♂, São Paulo, Serra da Cantareira, 900m, xii.1940. L. Travassos leg. (USNMENT 01222699, holotype#7,675).

***Ischnotoma* (*Icriomastax*) *zikani* (Alexander, 1936).** BRAZIL: 1♀, Rio de Janeiro, Campo Bello [Itatiaia], 700-800m, 22.xi.1933. Zikan leg. (USNMENT 01222592, holotype#5,935).

***Ischnotoma* (*Ischnotoma*) *araucana* (Alexander, 1929).** CHILE: 1♂, Curacautin, 14.xii.1950. Peña leg. (USNMENT 01222556).

***Ischnotoma* (*Ischnotoma*) *concinna* (Philippi, 1866).** CHILE: 1♂, Curanipe 4.xii.1953. Peña leg. (USNMENT 01222558).

***Ischnotoma* (*Ischnotoma*) *decorata* (Philippi, 1866).** CHILE: 1♂, Santiago, Marga Marga 7.x.1927. Jaffuel & Pirion leg. (USNMENT 01222885).

***Ischnotoma* (*Ischnotoma*) *delpontei* (Alexander, 1929).** CHILE: 1♂, Ensenada, 14.x.1926. E. Del Ponte leg. (USNMENT 01222481, holotype#3,938).

***Ischnotoma* (*Ischnotoma*) *eburnea* (Walker, 1848).** AUSTRALIA: 1♂, Tasmania, Montain Wellington, A. M. Lea leg. (USNMENT 01222914, #1,691).

***Ischnotoma* (*Ischnotoma*) *episema* Alexander, 1924.** AUSTRALIA: 1♀, New South Wales, Montain Kosciusko, 7.xii.1922, Goldfinch leg. (USNMENT 01222965, paratype#2,698).

***Ischnotoma* (*Ischnotoma*) *fagetorum* (Alexander, 1929):** ARGENTINA: 1♂, Patagonia, Bariloche, 28.xi-1.xii.1926. M. Edwards leg. (USNMENT 01222960, paratype#3,940).

***Ischnotoma* (*Ischnotoma*) *fastidiosa* (Skuse, 1890).** AUSTRALIA: 1♂, New South Wales, Wentworth Falls, 20.x.1930. Wilson leg. (USNMENT 01222962, #2,387).

***Ischnotoma* (*Ischnotoma*) *fuscostigmosa* (Alexander, 1929).** CHILE: 1♂, Chiloe Island, Dalcahue, 10-13.ii.1954. Peña leg. (USNMENT 01222724, #3,939).

***Ischnotoma* (*Ischnotoma*) *goldfinchi* Alexander, 1924.** AUSTRALIA: 1♀, New South Wales, Montain Kosciusko, 7.xii.1922, Goldfinch leg. (USNMENT 01222946, allotype#2,790).

***Ischnotoma* (*Ischnotoma*) *immaculipennis* Alexander, 1924.** AUSTRALIA: 1♀, New South Wales, Blue Montains, 22.i.1922, E. W. Ferguson leg. (USNMENT 01222445, holotype#2,697).

***Ischnotoma* (*Ischnotoma*) *larotypa* (Alexander, 1929).** CHILE: 1♀, Chiloe Island, Castro, 19.xii.1926. R. & E. Shannon leg. (USNMENT 01222735, holotype#3,941).

***Ischnotoma* (*Ischnotoma*) *par* (Walker, 1856).** AUSTRALIA: 1♀, Victoria, Millgrove. 1.iv.1928. F. E. Wilson leg. (USNMENT 01222933, #1,689).

***Ischnotoma* (*Ischnotoma*) *penai* (Alexander, 1952).** CHILE: 1♂, Chiloe Island, Aucar, 6-15.i.1952. L. E. Peña leg. (USNMENT 01222690, holotype of *I.* (*Isc.*) *guzmani*#9,551).

***Ischnotoma* (*Ischnotoma*) *peracuta* Alexander, 1971.** CHILE: 1♂, Llanquihue, Hornohuinco, xii.1968. Peña leg. (USNMENT 01222685, holotype#12,630).

***Ischnotoma* (*Ischnotoma*) *porteri* (Alexander, 1929).** CHILE: 1♂, Cautin, Villarrica, 30km NE, 16-31.xii.1969. Peña leg. (USNMENT 01222926, #3,942).

***Ischnotoma* (*Ischnotoma*) *postnotalis* (Alexander, 1929).** ARGENTINA: 1♂, Patagonia, Bariloche, 28.xi-1.xii.1926. M. Edwards leg. (USNMENT 01222927, paratype#3,943).

***Ischnotoma* (*Ischnotoma*) *prionoceroides* Alexander, 1922.** AUSTRALIA: 1♀, Tasmania, Cradle Valley, 3500 ft. 27.i.1923. Tonnoir leg. (USNMENT 01222707, #1,741).

***Ischnotoma* (*Ischnotoma*) *problematica* (Alexander, 1945).** CHILE: 1♀, Guape, 25.i.1943. Bullock leg. (USNMENT 01222786, holotype#7,678).

***Ischnotoma* (*Ischnotoma*) *rubriventris* (Macquart, 1846).** AUSTRALIA: 1♂, New South Wales, Dorrigo, 14.iii.1928. Heron leg. (USNMENT 01222924, #1,690).

Ischnotoma **(*Ischnotoma*)** ***rubroabdominalis*** **Alexander, 1922.** AUSTRALIA: 1♂, Tasmania, Waratah. Lea & Carter leg. (USNMENT 01222922, paratype#1,743).

Ischnotoma **(*Ischnotoma*)** ***rufistigmosa*** **(Macquart, 1838).** CHILE: 1♀, 1912. E. C. Reed leg (USNMENT 01222897).

Ischnotoma **(*Ischnotoma*)** ***rufiventris*** **(Macquart, 1838).** AUSTRALIA: 1♀, New South Wales, Barrington Tops, i.1925, S. U. Zool. Exp. leg. (USNMENT 01222906, paratype#3,770).

Ischnotoma **(*Ischnotoma*)** ***schneriana*** **(Alexander, 1928).** CHILE: 1♂, Marga Marga. A. Pirion leg. (USNMENT 01222799, holotype#3,944).

Ischnotoma **(*Ischnotoma*)** ***scutellumnigrum*** **Alexander, 1924.** AUSTRALIA: 1♀, New South Wales, Montain Kosciusko, 5.xii.1921. E. W. Ferguson leg. (USNMENT 01222917, allotype#2,699).

Ischnotoma **(*Ischnotoma*)** ***shannoniana*** **(Alexander, 1929).** CHILE: 1♂, Chiloe Island. Ancud, 20.i.1952, Peña leg. (USNMENT 01222913, #3,945).

Ischnotoma **(*Ischnotoma*)** ***silvai*** **(Alexander, 1929).** CHILE: 1♂, Malleco, Nahuelbuta, 800m 23-24.i.1977, L. Peña leg. (USNMENT 01222910, #3,946).

Ischnotoma **(*Ischnotoma*)** ***skuseana*** **Alexander, 1928.** AUSTRALIA: 1♂, Tasmania, Fern Tree, Mountain Wellington, 11.xi.1922, A. Tonnoir leg. (USNMENT 01222909, paratype#3,366).

Ischnotoma **(*Ischnotoma*)** ***tarwinensis*** **Alexander, 1928.** AUSTRALIA: 1♂, Victoria, Lower Tarwin, 22.xi.1925, G. F. Hizz leg. (USNMENT 01222816, holotype#3,367).

Ischnotoma **(*Ischnotoma*)** ***terminata*** **Alexander, 1928.** AUSTRALIA: 1♀, Tasmania, Mountain Wellington, 30.xi.1922, A. Tonnoir leg. (USNMENT 01222903, paratype#3,368).

***Ischnotoma* (*Ischnotoma*) *trunculata* (Alexander, 1962):** ARGENTINA: 1♂, Neuquen, Lago Lacar, 650m, 23.i.1954. Schachovsky leg. (USNMENT 01222819, holotyoe#11,089).

***Ischnotoma* (*Neotipula*) *maya* (Alexander, 1912).** GUATEMALA: 1♀, Aguná, 1030m, 16.viii.1902. G. Eisen leg. (USNMENT 01222401, holotype).

***Ischnotoma* (*Neotipula*) *paprzyckii* (Alexander, 1941).** PERU: 1♀, Satipo, 2.v.1939. Paprzycki leg. (USNMENT 01222403, holotype).

***Ischnotoma* (*Neotipula*) *pectinella* (Alexander, 1940).** PANAMA: 1♂, Potrerillos, 3000m, 20.v.1935. J. W. Macswain leg. (USNMENT 01222402, holotype).

***Ischnotoma* (*Neotipula*) *penata* (Alexander, 1966).** ECUADOR: 1♀, Quito, 9350m, 31.v.1963. L. Peña leg. (USNMENT 01222688, holotype #11,663).
